# Improving antibiotic utilization through an outpatient stewardship initiative

**DOI:** 10.1017/ash.2022.367

**Published:** 2023-07-10

**Authors:** Kayla M. Hiryak, Geena A. Kludjian, Jason C. Gallagher, Marissa J. Cavaretta

**Affiliations:** 1 Population Health/Enabling Services, Lehigh Valley Health Network, Allentown, Pennsylvania; 2 Department of Pharmacy, Cooper University Hospital, Camden, New Jersey; 3 Department of Pharmacy Practice, Temple University School of Pharmacy, Philadelphia, Pennsylvania

## Abstract

Antibiotic prescribing errors at hospital discharge are common. We designed a pharmacist-driven antimicrobial stewardship program to evaluate prescriptions prior to being transmitted to community pharmacies. Drug-related problems were identified in prescriptions for 48 of 149 patients, resulting in 55 interventions. Review at discharge improves outpatient prescribing of antimicrobials.

Medication errors are common during times of care transition, such as antibiotics prescribed on hospital discharge.^1,2^ These errors predispose patients to antimicrobial-resistant infections and drug toxicities.^3^ Antimicrobial stewardship programs (ASPs) reduce inappropriate antibiotic use, improving resistance rates, and optimizing healthcare outcomes,^4^ and although the benefits of these programs are favorable, the impact has largely been demonstrated in institutional settings.^3,4^ Patients complete more than one-third of their antibiotic course after they are discharged from the hospital.^3,5^ The quantity prescribed at discharge often exceeds guideline recommended durations by ∼50%^6,7^ for common infections such as community- acquired pneumonia, skin and soft-tissue infections, urinary tract infections, pyelonephritis, and uncomplicated intra-abdominal infections.^5,6^

A previous evaluation of patients at our institution showed that patients at discharge were prescribed antibiotic durations for a mean of 4.3 days beyond guideline recommendations.^8^ We hypothesized that pharmacists reviewing antibiotic orders at the time of discharge would improve antibiotic durations while allowing other potential prescriptive issues to be corrected. Here, we describe the impact of pharmacist review on discharge antimicrobial prescriptions.

## Methods

**Fig. 1.**
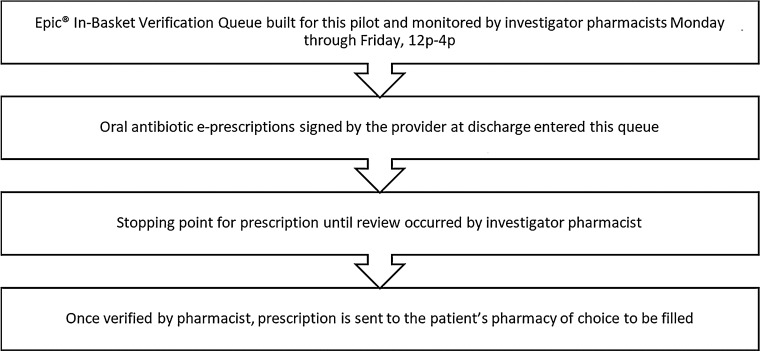
Verification queue pharmacist workflow.

This intervention was conducted at Temple University Hospital, a 722-bed academic medical center in Philadelphia, Pennsylvania. To identify patients discharged on oral antibiotics in real-time, a verification queue was built in the electronic health record (EHR; Epic Systems, Verona, WI) system (Fig. 1). Investigator pharmacists were second-year postgraduate residents who received similar training by institution infectious diseases (ID) and stewardship pharmacists. They reviewed discharge oral antibiotics prescriptions before the prescriptions were electronically sent to outpatient pharmacies. Prescriptions were reviewed Monday through Friday between 12 p.m. and 4 p.m. Outside this period, the discharge verification queue was inactive and defaulted back to the standard process for e-prescribing.

This project included patients in medicine wards who were discharged on oral antibiotics between February and May 2021. We applied the following exclusion criteria: patients who were pregnant, who were aged <18 years of age, who were diagnosed with COVID-19 infection, who were discharged on intravenous antibiotics, or who underwent patient-directed discharge. The primary outcome was the number of pharmacist interventions on oral antibiotic discharge prescriptions. Secondary outcomes included impact of pharmacist intervention on antibiotic duration compared to recommended guidelines, and cost savings.

Each prescription was reviewed for correct dose, frequency, duration, and safety using inpatient laboratory and procedure results, progress notes, and medication administration records. Appropriateness of antibiotic choice and duration was determined by evaluations of culture results when available, guidelines for empiric therapy, and both IDSA guideline and randomized control trial shortest-duration recommendations. Dosing was guided by institutional-based standards. Interventions were categorized as outlined in Table 1. Investigational pharmacists were not involved in prior authorization activities.

**Table 1.** Breakdown of Pharmacist Interventions

Intervention Type	No. (%)	Intervention Acceptance Rate, No. (%)
Total interventions	55	42 (76.4)
Inappropriate duration of therapy	19 (35.4)	11 (57.9)
**Incorrect dosing**	16 (29.1)	16 (100)
Underdosed	8	
Overdosed	7	
No directions	1	
**Antibiotic selection**	9 (16.4)	8 (88.9)
Organism resistant to prescribed antibiotic	3	
*Escherichia coli*	2	
*Staphylococcus aureus*	1	
Duplicate coverage	2	
Additional drug required	3	
Antibiotic streamlining	1	
Additional monitoring required	4 (7.3)	4 (100)
No indication for antibiotics	4 (7.3)	0 (0)
Drug–drug interaction	3 (4.4)	3 (100)

Data were collected on incidence, type, and acceptance rate of interventions. A cost-savings analysis was performed with values calculated by the EHR system (EPIC Hyperspace). Descriptive statistics were used.

## Results

In total, 149 patients had oral antibiotic prescriptions reviewed during the study period. Among them, 87 patients (58.4%) were male and 48 (32.2%) had at least 1 prescription with pharmacist intervention. Overall, 55 interventions were made and 76% of these were accepted.

Interventions are described in Table 1. Recommendations to shorten the duration of antibiotic therapy were the most common intervention (N = 19). The infections for which shorter durations were recommended were skin and skin-structure infections (median, 4.5 fewer days), lower respiratory tract infections (3 fewer days), complicated urinary tract infections (4 fewer days), and intra-abdominal infections (5 fewer days). Some interventions pertained to antibiotic selection and required therapy modification. Reasons for modification included demonstrated resistance to chosen antibiotic, inadequate empiric therapy, duplicate coverage, and antibiotic streamlining. Incorrect dosing resulted in 16 interventions with 6 antibiotics requiring dosing adjustments for renal function. To prevent adverse drug events, electrocardiograms were recommended for patients on multiple QTc-prolonging medications or a relevant history. Drug interactions were identified that required modification to administration instructions or creation of monitoring plans. Four antibiotics lacked an indication.

In addition, 35 patients had ID consultation prior to discharge. These patients required nominally less intervention than patients without expert consultation, though this difference did not reach significance: 8 of 35 versus 47 of 114 (*P* = .07).

The median time for pharmacist review and verification was 10 minutes (IQR, 5–15). The total estimated cost savings was $20,743. Using the 149 total patients and median pharmacist time for review, the calculated savings was ∼$800 for every hour of pharmacist effort. The projected cost savings related to direct interventions made.

## Discussion

Direct pharmacist review of discharge oral antibiotics led to interventions to improve prescribing in ∼33% of prescriptions. The genesis of the program was to improve prescribed durations of antibiotic therapy. However, once pharmacist review of live prescriptions began, additional opportunities to improve care were discovered.

Pharmacists are important to the prescribing process, often intervening at the point of dispensing to prevent errors or improve prescriptions. In our study, most interventions required access to patient information that most community pharmacists lack when they assess prescriptions. Intervening before electronic prescription transfer improves the ability of pharmacists to make assessments.

To our knowledge, a program in which pharmacists review discharge prescriptions in a queue prior to patient discharge has not been described. Without direct review of the e-prescriptions, room for error remains between discussion of discharge antibiotic plan and the prescription at time of discharge. Recently, Henry Ford Health System published a model in which pharmacists reviewed patients with active antimicrobials and anticipated discharges, creating discharge plans to discuss with discharge providers. Discharge antimicrobial orders were entered by the pharmacist and cosigned by provider. Comparatively, the manipulation of the EMR in our model allowed for direct review of these patients without report filtering.^9^

This study had several limitations. Our service was offered from 12–4 p.m., Monday through Friday, and only for medicine services. Therefore, only a proportion of discharge prescriptions were reviewed. Human resources for this pilot project were limited and could be expanded with dedicated personnel. Investigational pharmacists could filter outpatient prescriptions; however, all pharmacists had access to the outpatient prescriptions in the verification queue alongside inpatient orders, and a few outpatient prescriptions were inadvertently verified by another pharmacist. Additionally, e-prescriptions that originated as an inpatient order were not captured in the verification basket and were directly sent to the outpatient pharmacy without verification. Due to this system issue, likely some oral antibiotic discharge prescriptions were missed and were not assessed. Clinicians who may implement similar programs must be aware of this potential issue. Finally, attributable cost savings are difficult to calculate, and we did not evaluate them further than the values reported by the EHR.

The findings of this pilot study demonstrate the benefit of direct review of a queue of antibiotic discharge prescriptions by a pharmacist. Unnecessary antibiotics and dosing errors were identified, likely preventing unwanted adverse effects and outcomes associated with inappropriate antibiotic use. Additionally, significant cost savings may be associated with discharge stewardship. Expansion of a pharmacy-driven antimicrobial discharge stewardship program is warranted to seek additional favorable outcomes for patients. Incorporating this model to review other high-risk medications at discharge is another opportunity for transitions of care pharmacists to explore.
